# Experimentally manipulating forest structure to mimic management strategies: effects on deadwood fungal diversity and decomposition

**DOI:** 10.1093/femsec/fiag011

**Published:** 2026-02-11

**Authors:** Bronwyn Lira Dyson, Vendula Brabcová, Petr Baldrian, Jörg Müller, Michael Junginger, Claus Bässler

**Affiliations:** Ecology of Fungi, University of Bayreuth, Universitätsstraße 30, 95440 Bayreuth, Germany; Laboratory of Environmental Microbiology, Institute of Microbiology of the Czech Academy of Sciences, Vídeňská 1083, 14200 Prague, Czechia; Laboratory of Environmental Microbiology, Institute of Microbiology of the Czech Academy of Sciences, Vídeňská 1083, 14200 Prague, Czechia; Field Station Fabrikschleichach, Department of Animal Ecology and Tropical Biology, Biocenter, Julius-Maximilians-Universität Würzburg, 96181 Rauhenebrach, Germany; Bavarian Forest National Park, Freyunger Str. 2, 94481 Grafenau, Germany; Field Station Fabrikschleichach, Department of Animal Ecology and Tropical Biology, Biocenter, Julius-Maximilians-Universität Würzburg, 96181 Rauhenebrach, Germany; Ecology of Fungi, University of Bayreuth, Universitätsstraße 30, 95440 Bayreuth, Germany; Bavarian Forest National Park, Freyunger Str. 2, 94481 Grafenau, Germany

**Keywords:** alpha diversity, beta diversity, mass loss, canopy, microclimate, deadwood heterogeneity

## Abstract

Deadwood fungi are extremely diverse and crucial for carbon turnover in forests. To achieve multifunctional forests, we need to better understand the relationships between diversity, management, and ecosystem processes. We tested the effects of forest structure, i.e. canopy cover and deadwood enrichment, on fungal diversity and mass loss of European beech and Scots pine. We additionally assessed the effects of fungal diversity on mass loss. We expected deadwood enrichment to better explain fungal diversity, while canopy cover, alongside fungal diversity, would best explain mass loss. Overall, host tree species was more important than forest structure in explaining diversity. Beech fungal diversity was higher under closed canopies, while pine fungal diversity increased with some types of deadwood enrichment. Surprisingly, beech mass loss was higher in stands without deadwood enrichment, but also where tree crowns were added. Pine mass loss was not affected by forest structure. Effects of fungal diversity on mass loss were significantly related to fungal community composition in pine. Our findings emphasize the need for diverse tree hosts at the forest landscape-scale. However, contrasting diversity and decomposition effects between host trees indicate that stand-scale management strategies should be tailored to tree species to maintain diversity and decomposition processes.

## Teaser

Experimental study of the effects of forest management, via canopy cover and deadwood enrichment, on fungal diversity and decomposition. (Treatments illustration modified from Schwarz et al., 2025).

## Introduction

Modern forestry aims for multifunctionality, i.e. the attempt to produce simultaneously several ecological, social, and economic benefits (Pohjanmies et al. 2021), but this can necessitate substantial trade-offs. One of the most important conflicts in forestry is the desire to maximize wood production while maintaining biodiversity (Borrass et al. 2017). To improve forest management, we need to understand the consequences of forest structure, on the one side, as well as the complex relationships between management, biodiversity, and ecosystem processes, on the other side.

Two key structural axes in temperate forest ecosystems that drive forest biodiversity and related processes are deadwood as a critical resource and habitat (Stokland et al. 2012) as well as microclimate (Kemppinen et al. 2024). First, many forest species, including threatened ones, depend on deadwood (Seibold et al. 2015). However, deadwood enrichment as a concept in multifunctional forestry is not straightforward; a myriad of different niches exists, due to deadwood variability (Bujoczek et al. 2021). Summarized across studies, the host identity (e.g. tree species), type (e.g. logs, snags, stumps), dimension [e.g. coarse woody debris (CWD) and fine woody debris (FWD)], and decomposition stage have been identified as the main drivers of saproxylic diversity (e.g. Ódor et al. 2006, Yang et al. 2021). Furthermore, senescent (habitat) trees are key structures for threatened forest species (Bütler et al. 2013). However, due to forest management intensity over centuries, these trees and the species depending on them have become rare (Jones et al. 2018). Second, numerous studies have provided evidence of the importance of canopy-mediated microclimate variability for forest biodiversity (e.g. Nadkarni et al. 2001, Nakamura et al. 2017). Canopy openings lead to on average higher temperatures (Schreiber et al. 2024) and greater temperature variation (Braziunas et al. 2025). Altogether, it is well known that the amount and variety of deadwood as well as microclimate conditions are important predictors of forest biodiversity. To better inform forest management and address the trade-off between timber extraction and the maintenance of forest biodiversity, we need orthogonal experimental set-ups at the stand-scale. These experiments overcome confounding effects (e.g. variable deadwood availability or canopy-mediated microclimate after logging or disturbance) and provide evidence regarding the relative importance of the complex factors driving forest biodiversity.

Decomposition involves carbon sequestration and nutrient turnover and as such is a critical forest ecosystem process (Stokland et al. 2012). The decomposition rate of deadwood in a forest stand might be directly affected by forest structure. For example, under extreme and variable temperature and moisture conditions in open canopies, deadwood might be subject to physical stress, leaching, and weathering causing disintegration (Gora et al. 2019). Perhaps less pronounced, deadwood in the surroundings might affect decomposition rates by modifying microclimate conditions (e.g. via moisture maintenance; Schreiber et al. 2025a). Furthermore, although our understanding of fungal diversity-deadwood decomposition relationships is limited (Runnel et al. 2025), we posit that the diversity of fungi is linked to decomposition based on the biodiversity-ecosystem functioning (BEF) theory (Ali 2023).

We implemented a real-world forest experiment in which we manipulated resources and canopy cover at the stand-level (50 m by 50 m). The independent structural deadwood enrichment features were: logs, snags, logs and snags together, tree crowns, and habitat trees. We further considered total tree removal (including stump and roots) as a treatment. As a control, we used patches in which the trees were removed but stumps were left—a standard forest management strategy in central and south-eastern Europe (Mason et al. 2022). As a further independent abiotic feature, we manipulated the canopy cover (i.e. “open” vs. “closed” canopy). We used wood-inhabiting fungi as a model system as this group is extremely diverse (Wu et al. 2019), relevant to forest conservation (Müller et al. 2007), many are dependent on old trees and deadwood (Yang et al. 2021), and because fungi are the key agents driving deadwood decomposition in temperate forest ecosystems (Tláskal et al. 2021).

We sampled the microbial community of standardized exposed deadwood objects of European beech (*Fagus sylvatica* L.) and Scots pine (*Pinus sylvestris* L.) via metabarcoding and determined mass loss between 2018 and 2021. *Fagus sylvatica* and *P. sylvestris* are two of the most common tree species in European production forests (FISE 2025). First, we hypothesize that the relative importance of deadwood enrichment, as habitat and food for fungi, will be greater than that of canopy cover for explaining alpha and beta diversity. Furthermore, diversity should increase with CWD (i.e. logs, snags, and habitat trees) and FWD (i.e. crowns) enrichment, relative to the control, due to increased resource amount and variability. Second, we hypothesize that both forest structure and fungal diversity will explain deadwood mass loss but that canopy cover will be more important than deadwood enrichment.

## Material and methods

### Study area, design, and sampling

**Figure 1:**
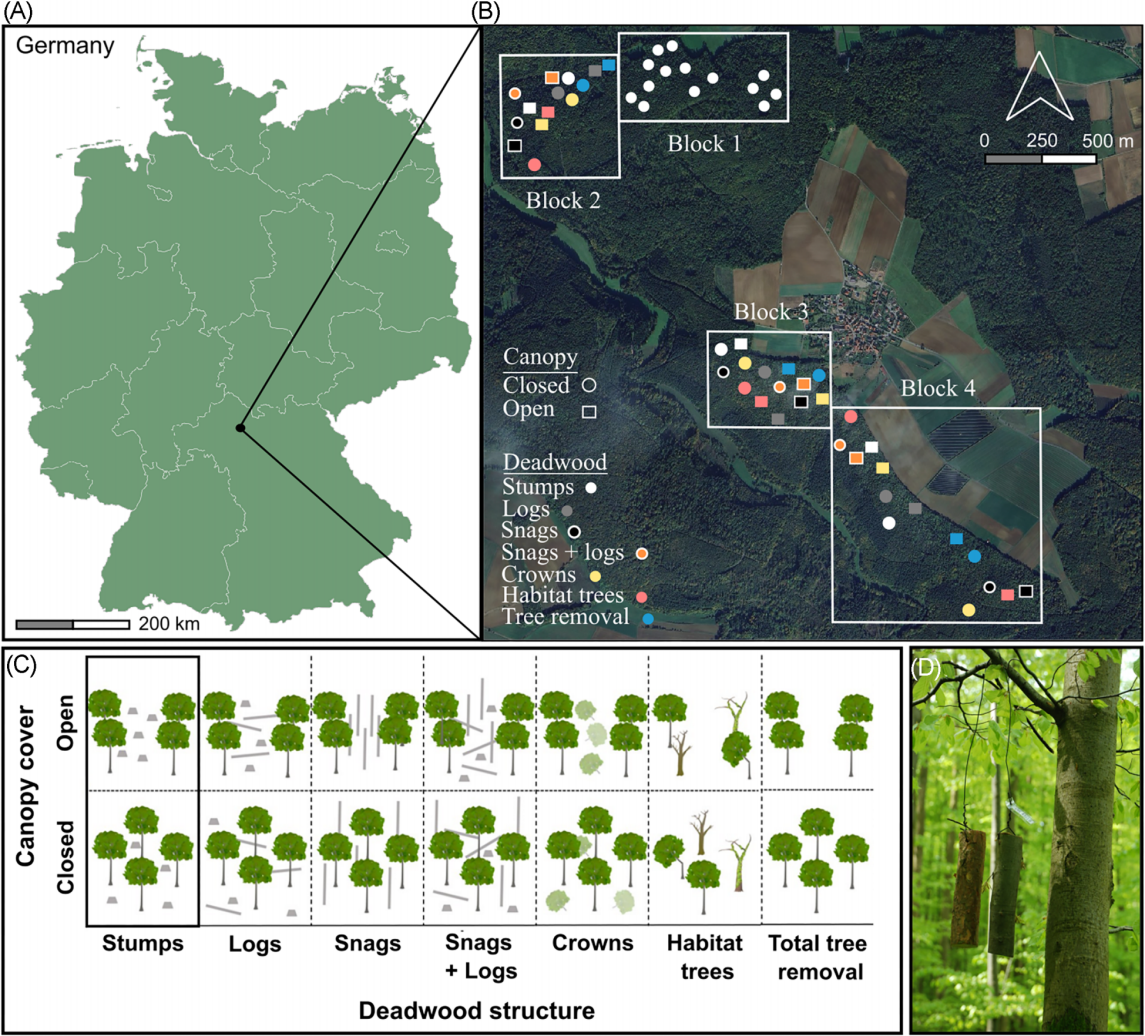
Overview of study area and experimental design. (a) Map of Germany with study location indicated. (b) A satellite map (© Google 2025) depicting part of the forest belonging to the Julius-Maximilians-University of Würzburg including the areas in which the study patches are located in their respective blocks as well as the town of Sailershausen. (c) Modified from Schwarz et al. (2025), an illustration of treatments where the top row of the illustration represents patches where the canopy is relatively open, due to the aggregated deadwood enrichment, and the bottom row represents patches where the canopy is relatively closed, due to the distributed deadwood enrichment. The control in our analyses was where stumps remain (black box). Patches were 50 m by 50 m. (d) A photo showing two pieces of standardized hanging wood per patch (*P. sylvestris* and *F. sylvatica*).

The study took place in the Julius-Maximilians-University of Würzburg forest (hereafter, “forest”) in Bavaria, Germany (Fig. 1). The forest (50.062°N, 10.444°E) is ∼2200 ha, intensively managed and mainly characterized by *F. sylvatica, Quercus* spp., *Carpinus betulus, Picea abies, P. sylvestris*, and other broad-leaf and coniferous tree species (Pierick and Ammer 2025). The average age of the stands was between ca. 80 and 100 years. The forest has a mean annual temperature of 8.5°C, annual precipitation of 670 mm (Stark et al. 2023), and an average elevation of 336 m.

Our study is part of a larger forest experimental framework (for more details, see Müller et al. 2023). The forest patches in our study were set up in 2018; we manipulated ca. 30% of the patch-level tree biomass at a scale of 50 m by 50 m (Kacic et al. 2024). The study patches were established across four forest blocks, hereafter “block”; whereas block one was a pure control block, blocks two–four contained a mixture of treatment and control patches (Fig. 1). In control patches we left only stumps, resembling the dominant tree harvesting practice in central European forests (Mason et al. 2022) (*n* = 20). The deadwood enrichment patches were as follows: (i) we left only logs and their associated stumps (*n* = 6), (ii) we left only snags (trees cut below the crown) (*n* = 6), (iii) we left logs and their associated stumps, and snags (*n* = 6), (iv) we left damaged trees (hereafter, “habitat” trees, i.e. with bark loss, damaged crowns, or tree hollows; Müller et al. 2014) (*n* = 6), (v) we removed trees but left tree crowns (*n* = 6), and (vi) trees, including stumps, were completely removed from the plots (*n* = 6). Deadwood enrichment consisted of broad-leaf and coniferous trees and was representative of stand tree species composition. The canopy cover treatments were: (i) aggregated deadwood enrichment manipulation, where we created canopy gaps by cutting trees in groups of ∼625 m² (*n* = 21) and (ii) distributed deadwood enrichment manipulation, where we avoided creating large canopy gaps by cutting trees spaced apart across the entire 50 m by 50 m patch (*n* = 35). We refer to the canopy treatments as “open” and “closed” hereafter.

As a standardized approach, we hung two logs of deadwood of two host trees, *F. sylvatica* and *P. sylvestris*, on each patch at the patch center either from tree branches or, where no trees were available, on wooden posts. The logs were not sterilized but were sourced from one stand in the forest. We used only trees without signs of damage. The logs were ∼15 cm long with a diameter of 6 cm. In 2018 and in 2021, we sawed an ∼4 cm slice from the hanging logs and froze them at −40°C. We used both slices to determine mass loss and the 2021 slice to analyze the microbial communities. Due to methodological restrictions, we could not include 13 of 112 mass loss data in the mass loss analyses (six data points from *F. sylvatica*, seven from *P. sylvestris*). For further details on the wood-drilling procedure and mass loss measurement see Supplementary Material 1.

### DNA extraction, PCR, amplicon sequencing, and OTU clustering

We used ∼200 mg of freeze-dried and milled sample to extract DNA via the NucleoSpin Bead Tube Type A (Macherey-Nagel, Germany). After adding Enhancer SX, we used an SL1 lysis buffer to lyse cells. We homogenized samples using FastPrep-24 (MP Biomedicals, USA) at 6.5 m s^−1^ for 2 × 30 s (Baldrian et al. 2016). Finally, we eluted DNA from columns using 50 μl of deionized water. DNA quality and quantity were assessed using a NanoDrop spectrophotometer (NanoDrop 2000, Thermo Scientific, USA). The fungal community composition was analyzed by sequencing the ITS2 region of DNA using barcoded primers gITS7 and ITS4 (Baldrian et al. 2016). To minimize bias, polymerase chain reaction (PCR) was performed in triplicate for each DNA sample. PCR triplicate reaction products were pooled and purified (MinElute PCR Purification Kit, Quiagen), and amplicon libraries prepared with the TruSeq DNA PCR-Free Kit LP (Illumina). Fungal amplicon sequencing and processing was conducted at the Institute of Microbiology at the Czech Academy of Sciences (Prague, Czechia). An Illumina MiSeq (2 × 250 base pair-end run) (Illumina, USA) was used, as described previously (Baldrian et al. 2016). Clustering of sequences into operational taxonomical units (OTUs) was done at a 97% similarity level. For simplicity, we refer to the OTUs hereafter as “species”. All sequencing data have been deposited in the NCBI SRA database under BioProject accession number PRJNA1310789. For further details regarding the PCR preparation and amplicon sequences processing, see Supplementary Material 2.

### Calculating alpha and beta diversity with the Hill numbers

All statistical analyses were performed using R Version 4.4.0 (R Core Team 2022). All samples had >2000 read sums and were thus all included in analyses. We estimated statistically the true number of singletons and calculated sample coverage-based diversity to avoid sampling bias effects in our analyses. For a more detailed description see Supplementary Material 3.

We used the Hill numbers, which can be understood as the effective or true diversities of communities (Hill 1973, Marion et al. 2021). An advantage to using the Hill numbers is the possibility it offers to assess distinct responses or patterns across abundance classes of species. The parameter *q* indicates how sensitive the index is to species abundances (Chao et al. 2014). For the alpha measure, *q* = 0 corresponds to species richness, where a disproportionate weight is given to rare species. *q* = 1 corresponds to Shannon diversity, where species are weighted proportionate to their abundances and common species are thus emphasized. *q* = 2 corresponds to the inverse of Simpson’s diversity, where dominant species are emphasized and rare species are discounted (Chao et al. 2014). For beta diversity, *q* = 0, 1, and 2 correspond to the Sørensen, Horn, and Morisita-Horn indices, respectively.

To calculate alpha diversity for *q* = 0, 1, and 2, we used the *estimadeD* function from the *iNEXT* package (Chao et al. 2014, Hsieh et al. 2016). To calculate beta diversity for the Hill numbers *q* = 0, 1, and 2, we used the *iNEXTbeta3D_pair3D* function (Chao et al. 2023, Kortmann et al. 2025). The result of this function is a sample coverage-based dissimilarity matrix for each *q* = 0, 1, and 2. We then subjected each dissimilarity matrix (*q* = 0, 1, and 2) to a principal coordinates analysis (PCoA), using the *pco* function from the *ecodist* package (Goslee and Urban 2007). Next, we extracted the scores of the first and second axes from each PCoA as vector response variables representing beta diversity in our models. The axes positions, and thus the distances among the scores, represent the dissimilarity of communities among samples (Koyanagi et al. 2018, Kümmet et al. 2025). This allowed us to specify our models for alpha and beta diversity within the same statistical framework (see Supplementary Material 4 for function settings used for calculating alpha and beta diversity).

### Statistical models

To test our first hypothesis, we modeled alpha diversity in response to the treatments, using a negative binomial model with a log link (*glmmTMB* function and package; Brooks et al. 2017) for both tree species samples together (hereafter “overall” model) as well as each of the tree species separately (hereafter “host tree species” model). We ran separate models also for each order of *q*. The rounded alpha diversity estimate was the response with canopy cover and deadwood enrichment as fixed effects and with patch nested in block as random effects for the overall models and block as a random effect in the host tree species models.

For modeling beta diversity, we used the scores of the first and second axes (PC1 and PC2, respectively) from the PCoA as response variables in separate models, as described above. We ran overall models as well as host tree species models. Canopy cover and deadwood enrichment were fixed effects with patch nested in block as random effects for the overall models and block as a random effect in the host tree species models. We used linear mixed-effects models (*lmer* function, *lme4* package; Bates et al. 2015). We acknowledge that this approach does not use the full variance of the ordination, but we explain considerable variability within the first two axes (across tree species and orders of *q*) (Supplementary Fig. S1). We investigated further approaches for analyzing beta diversity to ensure the robustness of our approach (see Supplementary Material 5 for details).

To test our second hypothesis, we used beta regressions for assessing the effects of canopy cover, deadwood enrichment, and diversity on the proportion of mass loss in each of the host tree species separately and for each diversity estimate separately, i.e. alpha diversity and beta diversity (represented by PC1 and PC2). We log_10_-transformed alpha diversity. We used the *gam* function from *mgcv* package (Wood 2011) with family = “betar” and link = “logit” and we included block as a random effect. We report deviance explained as the models are from a non-Gaussian family and thus have non-normal errors (Wood 2011, Clark 2022). Similarly to the host tree species models, we additionally ran an overall model including host tree as a fixed effect.

To verify whether our unbalanced design (see section above, “Study area, design, and sampling”) introduced heteroscedasticity in the variance of the scaled residuals of our models, we applied the functions *simulateResiduals* and *testCategorical* from the *DHARMa* package (Hartig 2025) for each model. This exercise revealed significant effects based on Levene’s test for two out of 46 of our models. To check whether heteroscedasticity changed the initial inferences of these two models, we ran nonlinear mixed-effects models with the argument varIdent, which allows for heteroscedastic residual variances (*nlme* function and package; Pinheiro et al. 2025) and compared our initial model’s estimates, effect sizes, and Akaike’s information criterion (AIC) with those of the nonlinear model. This analysis revealed that our initial model inferences were unchanged and that the AIC varied only minorly (see Supplementary Material 6).

## Results

### Effects of forest structure and host tree species on fungal alpha diversity

**Figure 2:**
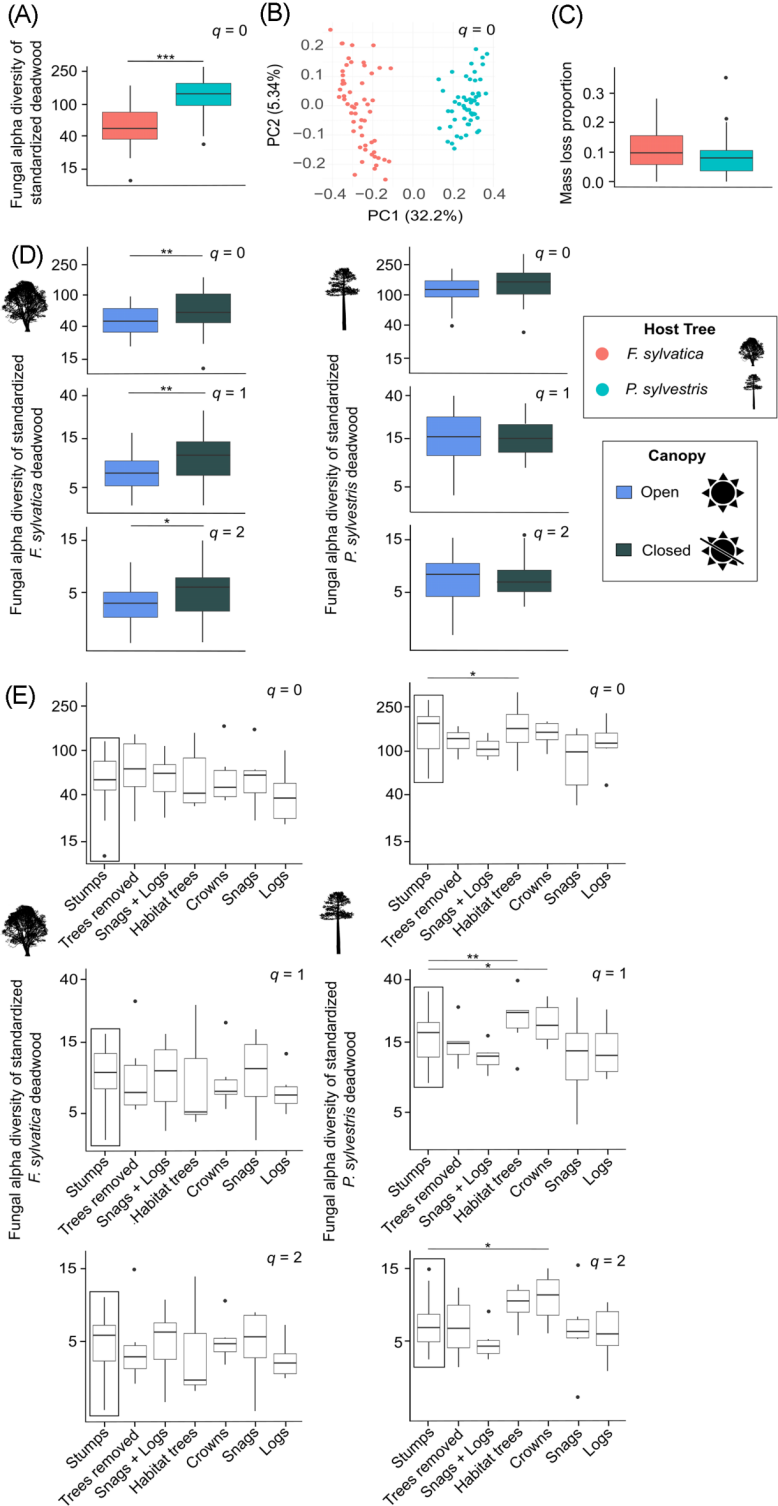
Host tree species effects on alpha diversity, as well as canopy and deadwood enrichment effects on alpha diversity, beta diversity, and mass loss. (a) Fungal species richness by host tree species (host effect: *z* = 9.224, *P* = <0.001, see Supplementary Table S1 for full model results). Significant results are indicated with asterisks (*P < 0.05, **P < 0.01, ***P < 0.001). (b) PCoA ordination plot illustrating the first (PC1) and second (PC2) axes for *q* = 0 for community composition analyses (host effect: *t* = 45.190, *P* = <0.001, see Supplementary Table S2 for full model results). (c) Mass loss proportion by host tree species (host effect: *z* = −1.831, *P* = 0.07). Fungal alpha diversity across orders of *q* for *F. sylvatica* and *P. sylvestris* host tree standardized deadwood in response to the canopy cover (d) and deadwood enrichment (e). Diversity values were log_10_-transformed for plotting and were back-transformed for display on the *y*-axis and are thus real alpha diversity estimates (i.e. effective number of species) that can be compared across host tree species and treatments. The control for the comparisons across deadwood enrichment were patches with stumps remaining (boxed in black). Contrasts to the control that were significant from the host tree species specific models (see Supplementary Table S1 for complete results) are indicated with asterisks (*P < 0.05, **P < 0.01, ***P < 0.001).

Among all model predictors, host tree species showed the strongest effect on alpha diversity for all orders of *q* (Supplementary Table S1). In the overall models, alpha diversity was consistently higher on *P. sylvestris* than on *F. sylvatica* (Fig. 2). The relative importance of deadwood enrichment and canopy cover on alpha diversity differed among orders of *q*. While for the diversity of rare species the effect of canopy cover was significant, the deadwood enrichment treatments were not significant for any abundance class of species in the overall models.

When modeling the response of alpha diversity for each of the host tree species separately, for *F. sylvatica*, fungal diversity was consistently (i.e. across all orders of *q*) higher in patches where the canopy was closed (Fig. 2). The fungal diversity of *P. sylvestris* was higher in patches where habitat trees were present, in terms of rare and common species, and where tree crowns were present, in terms of common and dominant species (Fig. 2). However, regarding the rare species of *P. sylvestris*, the effect of habitat trees is not visible in the raw plots and we therefore do not interpret this effect.

### Effects of forest structure and host tree species on fungal beta diversity

Similar to fungal alpha diversity, fungal beta diversity was mainly related to host tree species across all orders of *q* (Supplementary Table S2, Fig. 2), indicating that the community composition differs significantly between the host tree species. Furthermore, canopy cover was more important than deadwood enrichment in explaining variability in community composition. Specifically, the community composition of rare species for *F. sylvatica* differed significantly between open and closed canopies (Supplementary Table S2). This is clearly supported by the ordination (Supplementary Fig. S1). We found only one significant effect of deadwood enrichment on the community composition; for *P. sylvestris*, the community composition of rare species differed between patches with tree crowns and stumps. The amount of variance explained for PC1 of *F. sylvatica* was ca. 12%–30% and for PC2 7%–27% (Supplementary Fig. S1). The range in variance explained for PC1 of *P. sylvestris* was ca. 13%–25% and for PC2 9%–20% (Supplementary Fig. S1).

### Effects of forest structure and host tree species on mass loss

**Figure 3:**
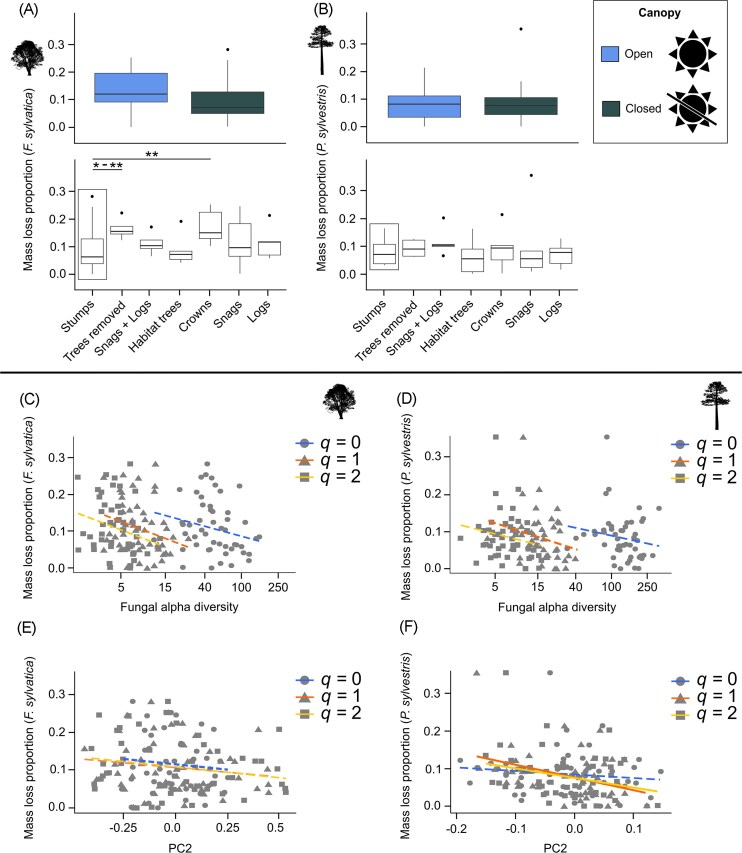
Canopy, deadwood enrichment, and diversity effects on mass loss. Proportional mass loss after three years for *F. sylvatica* (a) and *P. sylvestris* (b) in response to canopy and deadwood treatment. The control for the comparisons among deadwood was patches with stumps remaining (boxed in black). Contrasts to the control that were significant are indicated with asterisks (*P < 0.05, **P < 0.01, ***P < 0.001). Note, since nine models were run per host tree species, significance is depicted as a range where models resulted in different *P* values. Linear regressions of mass loss proportion as a function of alpha diversity for species richness for *F. sylvatica* (c) and *P. sylvestris* (d). Linear regressions of mass loss proportion as a function of community composition represented by PC2 for *F. sylvatica* (e) and for *P. sylvestris* (f). Solid lines indicate significant slopes, see Supplementary Table S3 for further details.

The effect of host tree species on mass loss was not significant in the overall model (Fig. 2). In the host tree species models, we only considered predictors significant when having significant effects across all beta regression models (i.e. across orders of *q* and diversity estimates). In the *F. sylvatica* models, neither canopy cover, alpha, nor beta diversity had significant effects on mass loss (Supplementary Table S3). Although the trend in alpha diversity was consistently negative (Fig. 3). Across orders of *q* and including models of each diversity estimate, there was significantly higher mass loss in patches with trees removed or with tree crowns compared to in control patches where only stumps remained (Supplementary Table S3, Fig. 3). As with *F. sylvatica*, the beta regressions revealed that although the trend was negative, alpha diversity was not a significant predictor of mass loss in *P. sylvestris*. We found a significant relationship between community composition and mass loss in *P. sylvestris* for *q* = 1 and *q* = 2 (Supplementary Table S3, Fig. 3).

## Discussion

Our large-scale experiment revealed that the host tree identity was a far better predictor of fungal diversity in deadwood after 3 years of decomposition than forest structure. Deadwood enrichment was more important than canopy cover for the fungal diversity of *P. sylvestris* although we found the opposite to be the case for *F. sylvatica* and thus, our first hypothesis is partially supported. Canopy cover was not significantly related to deadwood mass loss, in contrast to our second hypothesis. Instead, deadwood enrichment affected the mass loss of *F. sylvatica*. Mass loss was significantly related to the diversity of *P. sylvestris* and thus, our second hypothesis is partially supported. Overall, we found that the diversity and mass loss response was strongly host tree species specific.

### Effects of forest structure and host tree species on fungal alpha and beta diversity

Our overall models revealed that host tree species was more important for explaining fungal diversity than forest structure which, in our study, were represented by canopy cover and deadwood enrichment. This finding is in line with previous studies which demonstrated the importance of the host identity relative to abiotic factors, based on metabarcoding (Brabcová et al. 2022) and fruit bodies (Krah et al. 2018a), as well as in and across different macroclimate regimes (Rieker et al. 2022). We conclude therefore that this is a general phenomenon at the scale within and across landscapes in a biogeographic region. This might be because wood-inhabiting fungi and their hosts have coevolved across a broad range of environmental conditions in which the hosts could survive (Krah et al. 2018b).

Our study revealed that *P. sylvestris* was more diverse in species than *F. sylvatica*. This is in line with Purahong et al. (2018), who showed that coniferous logs hosted higher fungal alpha and gamma diversity than broad-leaf logs. Similarly, and even more relevant to our study due to comparable object size, Brabcová et al. (2022) found higher fungal alpha diversity in *Abies alba* than in *F. sylvatica* FWD. However, such findings contrast with Krah et al. (2018a), in which the authors found that *F. sylvatica* hosted higher fungal diversity than *A. alba*. One important difference in Krah et al. (2018a) is that the authors used fruit body inventories. In our study, in Purahong et al. (2018), and in Brabcová et al. (2022), metabarcoding data was used. These contrasting results may be explained by the prevalence of fruiting cues differing between broad-leaf and coniferous trees (Rieker et al. 2024). However, generalizing about diversity patterns based on the tree being an angio- or gymnosperm can be misleading. As Leonhardt et al. (2019) showed, fungal communities not only differed significantly between broad-leaf and coniferous hosts but also between species within each group. These fungal assembly patterns may be linked to variability in specific wood traits within (e.g. caused by environmental variability and growth conditions) and across tree species.

One further issue in comparing fungal diversity of broad-leaf and coniferous deadwood objects is that such a comparison after a defined time might simply reflect differences in successional (i.e. decomposition) stages. From previous studies, we know that deadwood succession is correlated with species richness (Rajala et al. 2010, Schreiber et al. 2024). By measuring mass loss in our study, we overcame this problem. As mass loss effects did not differ significantly between *F. sylvatica* and *P. sylvestris* and as *P. sylvestris* hosted on average approximately twice as many species as *F. sylvatica* (in terms of alpha diversity for rare and common species), we presume that *P. sylvestris* is more diverse, at least in this relatively early successional stage. Further studies are necessary to evaluate both diversity based on within-deadwood mycelia as well as the diversity of fruit bodies, standardized by decomposition over the course of succession, and considering tree species from different lineages.

Importantly, in both overall models for assessing the factors affecting alpha and beta fungal diversity, the effect size of the host tree species on diversity decreased between rare and dominant species (Tables S1 and S2). This may seem intuitive, but it has not often been explicitly tested and shown for fungal communities. This result may point to fungi that are abundant in deadwood being less host-dependent than rare fungi (Crisfield et al. 2024).

It is notable that canopy cover and deadwood enrichment affected fungal diversity differently depending on the host tree. First, fungal alpha diversity in *F. sylvatica* deadwood was higher under closed canopies. It may be that, in the case of *F. sylvatica*, the conditions under the closed canopy (generally cooler and wetter) were conducive to fungal establishment and growth. Second, fungal alpha diversity of *P. sylvestris* was higher on patches with habitat trees and tree crowns. This could be due to the “surface area effect” (Heilmann-Clausen and Christensen 2004). Assessing rarefaction curves, Heilmann-Clausen and Christensen (2004) found that small trees and branches hosted more fungal species per unit volume than larger trees and logs. Piché-Choquette et al. (2023) and Bässler et al. (2010), also found higher fungal diversity in FWD than in CWD. In both studies, the authors hypothesized that the higher habitat diversity (per volume) in FWD may explain the results. In FWD, there can be greater fluctuations in moisture and temperature, and, for branches and tree crowns, there could be more extreme fluctuations in humidity and light intensity (Shiegg 2001). Furthermore, habitat trees are extremely heterogenous as a fungal resource. These structures offer a combination of healthy and damaged bark, sapwood, and tree crown, as well as the combination of CWD and FWD (i.e. branches and remaining parts of the tree crown). Perhaps it is this niche heterogeneity at the patch-level that contributed to the heterogeneous fungal communities found in *P. sylvestris*.

This finding overall supports our expectation to find higher fungal diversity in patches with deadwood enrichment compared to those where only stumps remain, due to the increased availability and heterogeneity of deadwood resources. The fact that the variables explaining fungal diversity differed strongly between tree species deserves further attention. It remains unclear why canopy cover was important for explaining alpha diversity in *F. sylvatica* but not *P. sylvestris*. This might be related to the basic structure of forest habitats; *F. sylvatica*-dominated forests are rather darker than *P. sylvestris*-dominated forests (as light transmission in forests correlates to the shade-tolerance, successional status, and foliage clumping of the dominant species; Lintunen et al. 2013). Thus, fungal species associated with *F. sylvatica*-dominated forests may have evolved a stronger dependence on the relatively benign microclimate conditions of closed canopy forests compared to species associated with *P. sylvestris*-dominated forests.

### Effects of forest structure and host tree species on decomposition

In contrast to our expectation, mass loss was not affected by canopy cover but instead, in the case of *F. sylvatica*, by deadwood enrichment. Additionally, we found an effect of community composition on mass loss for *P. sylvestris*. In a recent study it was shown that after 10 years, CWD was more decomposed in open than closed canopies (Schreiber et al. 2025a). Furthermore, in that study, effects were more pronounced for *F. sylvatica* than *A. alba*, whereas in our case, we found that the broad-leaf and conifer hosts did not differ significantly in their mass loss (although on average, mass loss was highest in *F. sylvatica* under open canopies). Decomposition rates are related, e.g. to wood traits like lignin content, where higher concentrations and a denser structure of lignin inhibit the decomposition of coniferous wood (Hatakka and Hammel 2010). Perhaps the reason why we did not observe a significant relationship between mass loss and canopy cover nor between mass loss and tree species might be because our study covered the initial stages of decomposition.

We found that effects of deadwood enrichment were only significant and consistent across models for *F. sylvatica*. Mass loss of *F. sylvatica* deadwood was higher in forest patches where trees had been totally removed and where crowns remained. Effects from surrounding deadwood on mass loss might be caused by modified microclimate effects. Schreiber et al. (2025b) demonstrated that deadwood temperature was lower during the summer if the amount of surrounding deadwood was greater, suggesting buffering effects. Thus, the positive effect of crowns on *F. sylvatica* mass loss might be caused by benign temperature and moisture conditions allowing decomposer communities to increase their metabolic rate and decomposition capability (Schreiber et al. 2025a). However, it remains unclear why we did not observe similar effects with other types of deadwood enrichment and why mass loss was higher in patches where trees were totally removed. More studies are needed to evaluate the physical conditions mediated by deadwood enrichment that might modify metabolic rates of fungal communities adapted to different tree species.

We expected fungal diversity to be significantly related to wood mass loss following basic principles of BEF theory and as has been indicated by other studies that assessed the relationships between microbial diversity and decomposition (e.g. Jin et al. 2022, Ardestani et al. 2025). We observed significant effects of community composition on *P. sylvestris* mass loss. While Fukami et al. (2010) found a clear negative relationship between the number of species and decomposition, other studies observed opposite or weak relationships (Setälä and McLean 2004, Tiunov and Scheu 2005). Heilmann-Clausen and Boddy (2005) attribute observations of a negative relationship to antagonistic interactions among the many coexisting fungal species within wood. To improve our understanding of BEF relationships, we should focus on studying real-world assemblages in clearly defined environmental contexts (van der Plas 2019, Runnel et al. 2025), as our study does. We suggest that alpha diversity is less important than beta diversity in our BEF context. This is supported by studies which demonstrated that microbial community composition was sometimes as important as the number of species or more so, in driving ecosystem functions (e.g. Delgado-Baquerizo et al. 2017). One further interesting finding from our analysis was that the diversity effect (PC2) of *P. sylvestris* fungi on mass loss was stronger when weighted toward common and dominant species. Indeed, it has been suggested that abundant key decomposer species in deadwood are especially important for predicting the fungal diversity-decomposition relationship (Runnel et al. 2025). Further studies that consider not only the identity of fungal species but also their abundance and functional performance in a given environment are crucial to getting closer to understanding underlying BEF mechanisms.

## Conclusions

Using a large-scale field experiment manipulating canopy cover and deadwood enrichment, we show that wood-inhabiting fungal diversity and deadwood decomposition are shaped primarily by host tree species identity rather than forest structure. This underscores the importance of maintaining a broad range of host species across forest landscapes to sustain fungal diversity. At the same time, species-specific management strategies are crucial: in *F. sylvatica*-dominated forests, maintaining heterogeneous canopy cover promotes complementary fungal communities, whereas in *P. sylvestris*-dominated forests, fungal diversity benefits from deadwood enrichment, including the retention of tree crowns after logging and the protection or creation of habitat trees through premature-senescence strategies. Because deadwood mass loss is affected by both deadwood enrichment and fungal diversity, decomposition processes are highly sensitive to forest management, but strongly dependent on dominant tree species. To better predict biodiversity and ecosystem functioning outcomes, future research should expand across spatial and temporal scales and develop mechanistic insights into fungal community assembly, coexistence, and their functional roles in driving ecosystem processes.

## Supplementary Material

fiag011_Supplemental_File
