# Supplementary material for: Experimentally manipulating forest structure to mimic management strategies: effects on deadwood fungal diversity and decomposition
Source: FEMS Microbiol Ecol. 2026 Feb 11;102(3):fiag011. doi: 10.1093/femsec/fiag011 (PMC12927422; doi:10.1093/femsec/fiag011)
Supplement: fiag011_Supplemental_File [file fiag011_supplemental_file.docx]

**Supplementary Material**

This document contains Supporting Text which is intended to enhance the reader's understanding of our “Materials and methods” section. Below the Supporting Text, are the following Supplementary Figure and Tables: Figure S1 (ordination plot illustrating an aspect of the beta diversity analyses), Table S1 (containing the results from the negative binomial models that were run as part of the alpha diversity analyses), Table S2 (containing the results from the linear mixed effects models that were run as part of the beta diversity analyses), Table S3 (containing the results from the beta regression models that were run as part of the mass loss analyses).

**Supporting Text**

**1. Wood drilling procedure and mass loss measurement**

We defrosted the wood slices that we collected in 2018 and in 2021 directly before drilling for a sawdust sample. We used a drill (Makita, Japan) and an 8-mm diameter drill bit for collecting the deadwood sample. The drill bit was cleaned and sterilized with ethanol and flame before each slice was drilled. Two perpendicular drillings per slice were taken. We collected the sawdust directly into sterile plastic bags, which we froze immediately after drilling to conserve the DNA. The sawdust samples were then freeze-dried and milled using the Ultra Centrifugal Mill ZM 200 (Retsch, Germany).

We dried the wood slices from 2018 and 2021 at 50°C until the mass was constant. We removed fruit bodies that were present on a few slices. Next, we measured the dry mass of the slices with a fine scale (i.e. accurate to three decimal points). We then wrapped the wood slice in Parafilm (Bemis Company, USA) and determined its volume via water displacement using a glass volumetric vessel. To determine density, we divided the dry weight by the volume. Percent mass loss from 2018-2021 was calculated as follows:

((*dry weight/volume*^2018^ – *dry weight*/*volume*^2021^)/(*dry weight*/*volume*^2018^)) * 100

Of the 112 pairs of wood slices, we were unable to determine mass loss for 13 pairs. This affected six patches for *F. sylvatica* and seven patches for *P. sylvestris*. The specific treatments that were affected are given in Table 1 below:

**Table 1.** Patches excluded from mass loss analysis by host tree species and treatment.

| *F. sylvatica* | Deadwood enrichment | Canopy cover | *n* |
| --- | --- | --- | --- |
|  | Logs | Closed | 1 |
|  | Crowns | Closed | 1 |
|  | Trees removed | Closed | 2 |
|  | Snags + Logs | Open | 1 |
|  | Stumps | Closed | 1 |
| *P. sylvestris* | Snags | Open | 1 |
|  | Crowns | Closed | 1 |
|  | Stumps | Open | 1 |
|  | Snags + Logs | Open | 1 |
|  | Stumps | Closed | 3 |

**2. PCR preparation and amplicon sequence processing**

Each PCR contained 5 µl of 5× Q5 reaction buffer, 1.5 µl of BSA (10 mg ml-1), 1 µl of each primer (0.01 mM), 0.5 µl of PCR Nucleotide Mix (10 mM each), 0.25 µl of Q5 High Fidelity DNA polymerase (2 U µl -1, New England Biolabs, Inc.), 5 µl of 5× Q5 HighGC Enhancer and 1 µl of template DNA (approx. 50 ng µl-1). Cycling conditions were 98°C for 30 sec, 30 cycles of 94°C for 10 sec, 56°C for 30 sec, and 72°C for 30 sec, and a final extension at 72°C for 2 min. Negative and positive amplification controls were used to avoid contamination errors and to prove the efficiency of the PCR, but these controls were not sequenced within this project.

We processed the amplicon sequencing data using the pipeline SEED 2.1.3 (Větrovský & Baldrian 2013). We merged pair-end reads using fastq-join (Aronesty 2013) and extracted the ITS2 region with ITS Extractor 1.0.11 (Bengtsson-Palme et al. 2013). Using Usearch 11.0.667 (Edgar 2010), we detected chimeric sequences and removed them. Clustering of sequences into Operational Taxonomical Units (OTUs) was done using the UPARSE algorithm implemented within Usearch 8.1.1861 (Edgar 2013) at a 97% similarity level. The most abundant sequence was used to represent each OTU.

**3. Singletons correction and sample coverage**

Chiu & Chao’s (2016) approach for estimating the true number of singletons specifically modifies an OTU table by randomly setting singleton entries to zero such that the number of remaining singletons per sample is equal to the number of singletons estimated by Chiu & Chao’s method for sequencing error estimation. As this procedure involves a resampling approach, multiple runs (i.e. modified community matrices) should ideally be considered in the subsequent analysis. However, testing different resampled community matrices did not alter the inference of our final models. Therefore, for simplicity and reproducibility, we used *set.seed* (R Core Team 2022) and used a single representative community matrix after singleton correction.

Sampling bias or sample incompleteness is an issue in ecological research which can affect a study’s inferences (Chao & Jost 2012). For example, in our data, sampling bias can result from differences in detection probability (e.g. sequencing depth) caused by differences in environmental conditions (Kortmann et al. 2025). We calculated sample coverage using the *iNEXT* function and package (Chao et al. 2014a; Chao et al. 2014b; Hsieh et al. 2016) with datatype = “abundance”. We then continued by assessing potential biases in sample coverage in response to predictor variables using beta regression models, as sample coverage values are between 0 and 1. We used the *gam* function from the *mgcv* package (Wood 2011), specifying patch in block as nested random effects (family = “betar”, link = “logit”). The response variable was sample coverage and the fixed effects were tree species, canopy cover, and deadwood enrichment. Biases (i.e., statistically significant effects of predictors on sample coverage) would suggest that a standardized level of sample coverage for diversity analyses is necessary. We found that tree species had significant effects on sample coverage – indicating that standardization based on sample coverage was necessary for further analyses.

**4. Function settings for calculating alpha and beta diversity**

To calculate alpha diversity, we used the settings: datatype = “abundance”, base = “coverage”, level = NULL. When level = NULL, this is the default setting; the function computes the diversity estimates for the minimum among all the coverage values for samples extrapolated to double the reference sample size. To calculate beta diversity, we used the settings: datatype = “abundance”, SC = Cmax_joint. The input value for sample coverage (“Cmax_joint”), was computed as the minimum among all the coverage values for samples extrapolated to double the reference sample sizes (Chao et al. 2023).

**5. Modelling beta diversity**

To verify our selected approach of using the values of the first and second PCoA axes as vector responses in our beta diversity modelling, we applied additional models with up to four PCoA axes, we ran a PERMANOVA, and calculated environmental fits directly on to the ordination (function *envfit*, *vegan* package; Oksanen et al. 2025), but these approaches yielded no further insights (data not shown).

**6. Heteroscedasticity**

As explained in the main text (see “Study area, design, and sampling”), our experimental design is unbalanced due to the larger number of patches with stumps and patches with a closed canopy. Including block one (comprised solely of patches with stumps and closed canopy) allowed us to increase the sample size in our design and, specifically, the number of control patches, which should increase the power and robustness of our statistical analyses. On the other hand, the imbalance could lead to heteroscedasticity across levels of deadwood enrichment or canopy cover. Further, as outlined in “Statistical models” in the main text, we tested for heteroscedasticity in the variance across deadwood enrichment and canopy cover levels in each of our 46 models and we present the results here (Table 2, below).

**Table 2.** Results from Levene’s test (from the *DHARMa* R package, Hartig 2025) for assessing homogeneity of variance in the scaled residuals based on deadwood enrichment and canopy cover. Where bold *p*-values indicate a statistically significant result (i.e., heteroscedasticity is evident in the data).

|  | | | Deadwood enrichment | | Canopy cover | |
| --- | --- | --- | --- | --- | --- | --- |
|  | ***q*** | **Data subset** | **F value** | **Pr(>F)** | **F value** | **Pr(>F)** |
| Alpha | *q* = 0 | Overall | 0.377 | 0.892 | 0.489 | 0.486 |
|  | *q* = 1 | Overall | 0.636 | 0.701 | 0.530 | 0.468 |
|  | *q* = 2 | Overall | 0.531 | 0.784 | 0.075 | 0.785 |
|  | *q* = 0 | *F. sylvatica* | 0.296 | 0.936 | 3.941 | 0.052 |
|  | *q* = 1 | *F. sylvatica* | 0.198 | 0.976 | 0.450 | 0.505 |
|  | *q* = 2 | *F. sylvatica* | 0.276 | 0.946 | 1.105 | 0.298 |
|  | *q* = 0 | *P. sylvestris* | 0.757 | 0.607 | 0.244 | 0.624 |
|  | *q* = 1 | *P. sylvestris* | 1.290 | 0.280 | 0.009 | 0.927 |
|  | *q* = 2 | *P. sylvestris* | 0.422 | 0.862 | 0.068 | 0.795 |
| Beta | *q* = 0 | Overall | PC1: 0.785  PC2: 0.765 | 0.584  0.599 | 0.828  0.380 | 0.365  0.539 |
|  | *q* = 1 | Overall | PC1: 0.829  PC2: 0.532 | 0.550  0.783 | 0.033  0.088 | 0.857  0.767 |
|  | *q* = 2 | Overall | PC1: 0.789  PC2: 0.667 | 0.580  0.676 | 0.070  0.860 | 0.791  0.356 |
|  | *q* = 0 | *F. sylvatica* | PC1: 0.783  PC2: 0.472 | 0.588  0.826 | 4.619  2.904 | **0.036**  0.094 |
|  | *q* = 1 | *F. sylvatica* | PC1: 1.273  PC2: 0.633 | 0.287  0.704 | 0.123  9e-04 | 0.727  0.977 |
|  | *q* = 2 | *F. sylvatica* | PC1: 1.106  PC2: 1.703 | 0.373  0.140 | 0.482  0.895 | 0.491  0.349 |
|  | *q* = 0 | *P. sylvestris* | PC1: 0.191  PC2: 0.556 | 0.978  0.763 | 10.884  1.904 | **0.002**  0.173 |
|  | *q* = 1 | *P. sylvestris* | PC1: 0.581  PC2: 0.647 | 0.743  0.692 | 1.200  0.010 | 0.278  0.922 |
|  | *q* = 2 | *P. sylvestris* | PC1: 0.458  PC2: 0.427 | 0.836  0.858 | 0.497  0.013 | 0.484  0.908 |
| Mass loss | n.a. | Overall | 1.243 | 0.292 | 0.061 | 0.806 |
|  | *q* = 0 | *F. sylvatica* | Alpha: 0.674  PC1: 1.229  PC2: 1.142 | 0.671  0.312  0.355 | 0.142  0.033  0.026 | 0.708  0.856  0.874 |
|  | *q* = 1 | *F. sylvatica* | Alpha: 0.853  PC1: 1.079  PC2: 1.147 | 0.537  0.390  0.352 | 0.017  0.580  0.172 | 0.897  0.450  0.681 |
|  | *q* = 2 | *F. sylvatica* | Alpha: 0.798  PC1: 0.877  PC2: 1.150 | 0.577  0.520  0.351 | 0.069  0.321  0.061 | 0.794  0.573  0.806 |
|  | *q* = 0 | *P. sylvestris* | Alpha: 0.396  PC1: 0.454  PC2: 0.494 | 0.878  0.838  0.809 | 0.012  0.257  0.212 | 0.913  0.614  0.647 |
|  | *q* = 1 | *P. sylvestris* | Alpha: 0.375  PC1: 0.398  PC2: 0.251 | 0.891  0.876  0.956 | 0.009  0.009  1.513 | 0.924  0.924  0.225 |
|  | *q* = 2 | *P. sylvestris* | Alpha: 0.309  PC1: 0.293  PC2: 0.458 | 0.929  0.937  0.835 | 0.071  0.004  0.874 | 0.792  0.951  0.355 |

While there was no evidence of heteroscedasticity across deadwood enrichment levels, there were two instances for the canopy cover variable. In the case of the first instance of heteroscedasticity (i.e. beta diversity, *q* = 0, PC1, *F. sylvatica*), if we review our main analysis results (Table S2), we see that our finding (i.e. that there is a significant effect of canopy cover on community composition) holds for both the model in which heteroscedasticity was indicated (PC1) but also for the model in which heteroscedasticity was not evident (PC2). However, to ensure the robustness of our results, we applied an additional nonlinear mixed-effects model with the same construction as our initial models, with the exception that we used the varIdent argument (varIdent = canopy cover) to indicate that the variance function should allow for different variances based on canopy level (*nlme* function and package; Pinheiro et al. 2025). This model showed that canopy cover still had a significant effect on community composition (PC1), supporting the result of our initial model. Furthermore, the nonlinear model had only a slightly lower AIC than the initial model (ΔAIC ≈ 3). We therefore determine the result of our initial model to be robust. In the second instance of heteroscedasticity (i.e. beta diversity, *q* = 0, PC1, *P. sylvestris*), note that there was no significant relationship between canopy cover and beta diversity in the main model. Further, our initial model and the nonlinear model allowing heteroscedastic residual variances were essentially equivalent (ΔAIC ≈ 0.62); the heteroscedasticity did not change our initial results.

**References**

Aronesty, E. (2013). Comparison of Sequencing Utility Programs. *Open Bioinform. J.*, *7*(1), 1–8. https://doi.org/10.2174/1875036201307010001

Bengtsson-Palme, J., Ryberg, M., Hartmann, M., Branco, S., Wang, Z., Godhe, A., De Wit, P., Sánchez-García, M., Ebersberger, I., de Sousa, F., Amend, A. S., Jumpponen, A., Unterseher, M., Kristiansson, E., Abarenkov, K., Bertrand, Y. J. K., Sanli, K., Eriksson, K. M., Vik, U., Veldre, V., … Nilsson, R. H. (2013). Improved software detection and extraction of ITS1 and ITS2 from ribosomal ITS sequences of fungi and other eukaryotes for analysis of environmental sequencing data. *Methods Ecol. Evol., 4*(10), 914–919. https://doi.org/10.1111/2041-210X.12073

Chao, A., & Jost, L. (2012). Coverage-based rarefaction and extrapolation: standardizing samples by completeness rather than size. *Ecology, 93*(12), 2533-2547. https://doi.org/10.1890/11-1952.1

Chao, A., Gotelli, N. J., Hsieh, T. C., Sander, E. L., Ma, K. H., Colwell, R. K., & Ellison, A. M. (2014a). Rarefaction and extrapolation with Hill numbers: a framework for sampling and estimation in species diversity studies. *Ecol. Monogr.*, *84*(1), 45-67. https://doi.org/10.1890/13-0133.1

Chao, A., Chiu, C.‑H., & Jost, L. (2014b). Unifying Species Diversity, Phylogenetic Diversity, Functional Diversity, and Related Similarity and Differentiation Measures Through Hill Numbers. *Annu. Rev. Ecol. Evol. Syst.*, *45*(1), 297–324. https://doi.org/10.1146/annurev-ecolsys-120213-091540

Chao, A., Thorn, S., Chiu, C.‑H., Moyes, F., Hu, K.‑H., Chazdon, R. L., Wu, J., Magnago, L. F. S., Dornelas, M., Zelený, D., Colwell, R. K., & Magurran, A. E. (2023). Rarefaction and extrapolation with beta diversity under a framework of Hill numbers: The iNEXT.beta3D standardization. *Ecol. Monogr.*, *93*(4), e1588. https://doi.org/10.1002/ecm.1588

Chiu, C.‑H., & Chao, A. (2016). Estimating and comparing microbial diversity in the presence of sequencing errors. *PeerJ*, *4*, e1634. https://doi.org/10.7717/peerj.1634

Edgar, R. C. (2010). Search and clustering orders of magnitude faster than BLAST. *Bioinformatics*, *26*(19), 2460–2461. https://doi.org/10.1093/bioinformatics/btq461

Edgar, R. C. (2013). UPARSE: highly accurate OTU sequences from microbial amplicon reads. *Nat. Methods*, *10*, 996–998. https://doi.org/10.1038/nmeth.2604

Fox, J., & Weisberg, S. (2019). *An R Companion to Applied Regression* (3rd ed.). Sage.

Hartig, F. (2025). DHARMa: *Residual diagnostics for hierarchical (multi-level/mixed) regression models* (R package Version 0.4.7). [Computer software]. https://github.com/florianhartig/dharma

Hsieh, T. C., Ma, K. H., & Chao., A. (2016). iNEXT: an R package for rarefaction and extrapolation of species diversity (Hill numbers). *Methods Ecol. Evol.*, *7*, 1451–1456. https://doi:10.1111/2041-210X.12613

Koller, M. (2016). *robustlmm: Robust linear mixed effects models* (R package Version 2.5-0) [Computer software]. Comprehensive R Archive Network (CRAN). https://CRAN.R-project.org/package=robustlmm

Kortmann, M., Chao, A., Schaefer, H. M., Blüthgen, N., Gelis, R., Tremlett, C. J., Busse, A., Püls, M., Seibold, S., Kriegel, P., Rabl, D., La Hoz, M. de, Şekercioğlu, Ç. H., Schleuning, M., Feldhaar, H., Newell, F. L., Kümmet, S., Mitesser, O., Peters, M. K., & Müller, J. (2025). Sample coverage affects diversity measures of bird communities along a natural recovery gradient of abandoned agriculture in tropical lowland forests. *J. Appl. Ecol.*, *62*(3), 480–491. https://doi.org/10.1111/1365-2664.14879

Oksanen, J., Simpson, G., Blanchet, F., Kindt, R., Legendre, P., Minchin, P., O'Hara, R., Solymos, P., Stevens, M., Szoecs, E., Wagner, H., Barbour, M., Bedward, M., Bolker, B., Borcard, D., Borman, T., Carvalho, G., Chirico, M., De Caceres, M., Durand, S., … Weedon, J. (2025). *vegan: Community Ecology Package*. https://vegandevs.github.io/vegan/.

Pinheiro, J., Bates, D., & R Core Team. (2025). *nlme: Linear and nonlinear mixed effects models* (R package Version 3.1-168) [Computer software]. https://doi.org/10.32614/CRAN.package.nlme

R Core Team. (2022). *R: A language and environment for statistical computing*. R Foundation for Statistical Computing. https://www.R-project.org/

Větrovský, T., & Baldrian, P. (2013). Analysis of soil fungal communities by amplicon pyrosequencing: current approaches to data analysis and the introduction of the pipeline SEED. *Biol. Fertil. Soils.*, *49*(8), 1027–1037. https://doi.org/10.1007/s00374-013-0801-y

Wood, S. N. (2011). Fast stable direct fitting and smoothness selection for generalized additive models. *J. R. Stat. Soc. Ser. B Stat. Methodol.*, *73*(1), 3–36. https://doi.org/10.1111/j.1467-9868.2010.00749.x


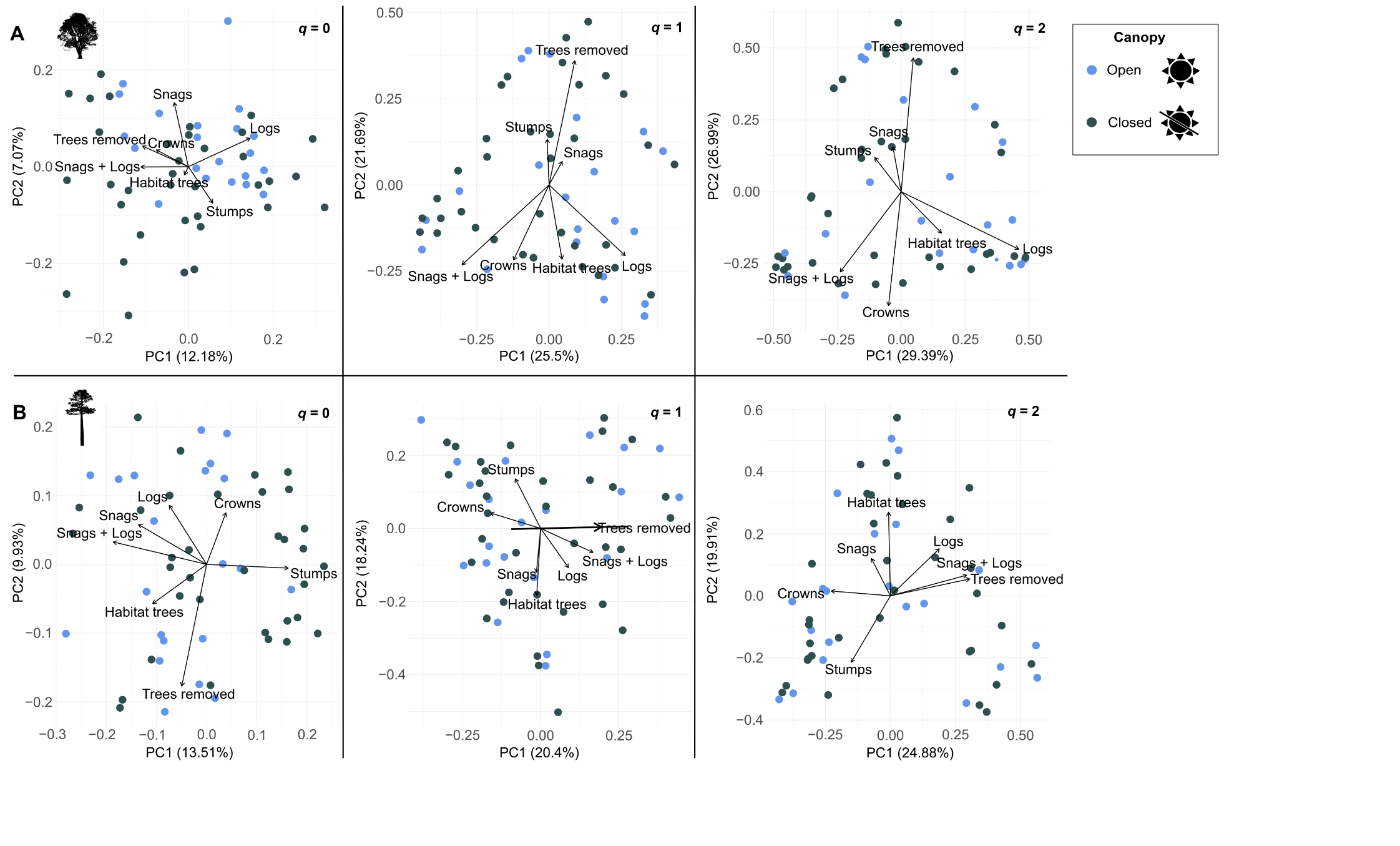


**Figure S1.** **Ordination plots of the relation of canopy and deadwood treatments to fungal beta diversity (i.e. community composition).** Ordinations are based on Principal Coordinate Analyses (PCoA) for *F. sylvatica* (A) and *P. sylvestris* (B) with canopy cover (colored points) and deadwood enrichment (arrows) indicated along the orders of *q*.

**Table S1.** Test statistics for fungal alpha diversity using negative binomial models with a log link. In the overall model, *F. sylvatica* and *P. sylvestris* samples were modelled together. Alpha diversity estimates were rounded before modelling. Patch nested in block were random effects for the overall model, block was a random effect in the host tree species models. Reference levels for canopy cover and host tree are indicated in parentheses. The reference level for deadwood enrichment was patches where only stumps were remaining. Significant effects are indicated as: * *p* < 0.05, ** *p* < 0.01, *** *p* < 0.001.

|  | Fixed effects | *q* = 0, rare | | | *q* = 1, common | | | *q* = 2, dominant | | |
| --- | --- | --- | --- | --- | --- | --- | --- | --- | --- | --- |
|  |  | **z value** | **Pr(>\|z\|)** | | **z value** | **Pr(>\|z\|)** | | **z value** | **Pr(>\|z\|)** | |
| Overall model | Canopy (Open) | **2.740** | **0.006**** | | 1.493 | 0.136 | | 1.482 | 0.138 | |
|  | Trees removed | 1.002 | 0.316 | | 0.548 | 0.584 | | 0.412 | 0.681 | |
|  | Snags + Logs | -0.082 | 0.935 | | -0.342 | 0.732 | | -0.665 | 0.506 | |
|  | Habitat trees | 1.416 | 0.157 | | 1.905 | 0.057 | | 1.404 | 0.160 | |
|  | Crowns | 0.879 | 0.379 | | 1.326 | 0.185 | | 1.952 | 0.051 | |
|  | Logs | -0.492 | 0.623 | | -0.648 | 0.517 | | -0.985 | 0.324 | |
|  | Snags | -0.032 | 0.975 | | 0.329 | 0.742 | | 0.305 | 0.760 | |
|  | Host tree (*F. sylvatica*) | **9.224** | **<0.001***** | | **6.338** | **<0.001***** | | **3.963** | **<0.001***** | |
|  | Marginal R^2^ | 0.482 | | | 0.325 | | | 0.213 | | |
| *F. sylvatica* | Canopy (Open) | **3.192** | | **0.001**** | **2.734** | | **0.006**** | **2.248** | | **0.025*** |
|  | Trees removed | 1.524 | | 0.128 | 0.922 | | 0.357 | 0.889 | | 0.374 |
|  | Snags + Logs | 0.689 | | 0.491 | 0.766 | | 0.444 | 0.605 | | 0.545 |
|  | Habitat trees | 0.631 | | 0.528 | 0.580 | | 0.562 | 0.425 | | 0.671 |
|  | Crowns | 0.869 | | 0.385 | 0.275 | | 0.783 | 0.444 | | 0.657 |
|  | Logs | -0.781 | | 0.435 | -0.506 | | 0.613 | -0.826 | | 0.409 |
|  | Snags | 0.922 | | 0.356 | 0.997 | | 0.319 | 0.597 | | 0.550 |
|  | Marginal R^2^ | 0.222 | | | 0.169 | | | 0.141 | | |
| *P. sylvestris* | Canopy (Open) | 0.265 | | 0.791 | -0.710 | | 0.477 | 0.005 | | 0.996 |
|  | Trees removed | 0.650 | | 0.516 | 0.470 | | 0.638 | 0.012 | | 0.990 |
|  | Snags + Logs | 0.018 | | 0.985 | -0.637 | | 0.524 | -1.243 | | 0.214 |
|  | Habitat trees | **2.098** | | **0.036*** | **2.659** | | **0.008**** | 1.671 | | 0.095 |
|  | Crowns | 1.273 | | 0.203 | **2.236** | | **0.025*** | **2.358** | | **0.018*** |
|  | Logs | 0.549 | | 0.583 | 0.027 | | 0.978 | -0.473 | | 0.637 |
|  | Snags | -0.591 | | 0.554 | 0.030 | | 0.976 | 0.129 | | 0.898 |
|  | Marginal R^2^ | 0.138 | | | 0.218 | | | 0.198 | | |

**Table S2.** Test statistics for beta diversity from fitting linear mixed-effects models to the values of the first and second axes (PC1 and PC2, representing community composition) of the PCoA for the overall model and for *F. sylvatica* and *P. sylvestris* specific models in response to canopy cover, deadwood enrichment, and (in the case of the overall model) host tree species. Reference levels for canopy cover and host tree are indicated in parentheses. The reference level for deadwood enrichment patches where only stumps were remaining. Significant effects are indicated in bold and as: * *p* < 0.05, ** *p* < 0.01, *** *p* < 0.001.

|  | Fixed effects | *q* = 0, rare | | | | | | | *q* = 1, common | | | | | *q* = 2, dominant | | | |
| --- | --- | --- | --- | --- | --- | --- | --- | --- | --- | --- | --- | --- | --- | --- | --- | --- | --- |
|  |  | **PC1** | | | **PC2** | | | | **PC1** | | **PC2** | | | **PC1** | | **PC2** | |
|  |  | **t value** | | **Pr(>\|t\|)** | **t value** | | **Pr(>\|t\|)** | | **t value** | **Pr(>\|t\|)** | **t value** | **Pr(>\|t\|)** | | **t value** | **Pr(>\|t\|)** | **t value** | **Pr(>\|t\|)** |
| Overall model | Canopy (Open) | 1.760 | | 0.082 | **-2.501** | | **0.016**** | | 1.548 | 0.125 | -0.735 | 0.464 | | 1.473 | 0.147 | 1.282 | 0.203 |
|  | Trees removed | 1.537 | | 0.127 | -0.266 | | 0.792 | | 0.644 | 0.522 | 0.391 | 0.697 | | 0.364 | 0.718 | -0.444 | 0.659 |
|  | Snags + Logs | -1.446 | | 0.151 | -1.555 | | 0.127 | | 0.349 | 0.728 | -1.432 | 0.157 | | 0.560 | 0.579 | 0.905 | 0.369 |
|  | Habitat trees | -1.357 | | 0.178 | -1.789 | | 0.080 | | 0.524 | 0.603 | -0.208 | 0.836 | | 0.445 | 0.659 | -0.541 | 0.590 |
|  | Crowns | -1.575 | | 0.118 | -1.11 | | 0.272 | | -0.501 | 0.619 | -0.793 | 0.431 | | -0.060 | 0.953 | 0.276 | 0.783 |
|  | Logs | -1.755 | | 0.082 | -0.016 | | 0.987 | | -0.424 | 0.673 | 0.919 | 0.361 | | -0.465 | 0.645 | -1.441 | 0.154 |
|  | Snags | -0.692 | | 0.491 | -0.311 | | 0.757 | | 0.687 | 0.495 | 0.001 | 0.999 | | 1.191 | 0.241 | -0.190 | 0.850 |
|  | Host tree (*F. sylvatica*) | **45.190** | | **<0.001***** | 0.862 | | 0.393 | | **45.789** | **<0.001***** | 0.654 | 0.514 | | **31.955** | **<0.001***** | -1.068 | 0.288 |
|  | Marginal R^2^ | 0.949 | | | 0.096 | | | | 0.950 | | 0.057 | | | 0.896 | | 0.073 | |
| *F. sylvatica* | Canopy (Open) | **-2.237** | | **0.030*** | **-2.206** | **0.032*** | | | -1.419 | 0.162 | 0.302 | 0.764 | | -1.575 | 0.122 | -0.110 | 0.913 |
|  | Trees removed | -1.089 | | 0.282 | 0.766 | 0.448 | | | 0.101 | 0.920 | 1.403 | 0.168 | | 0.196 | 0.846 | 1.466 | 0.151 |
|  | Snags + Logs | -1.116 | | 0.271 | 0.372 | 0.712 | | | -1.568 | 0.124 | -1.385 | 0.174 | | -0.803 | 0.427 | -1.227 | 0.227 |
|  | Habitat trees | -0.432 | | 0.668 | 0.222 | 0.826 | | | -0.088 | 0.930 | -1.310 | 0.198 | | 0.582 | 0.564 | -0.738 | 0.465 |
|  | Crowns | -0.874 | | 0.387 | 0.694 | 0.491 | | | -0.807 | 0.424 | -1.323 | 0.193 | | -0.142 | 0.887 | -1.653 | 0.106 |
|  | Logs | 0.603 | | 0.550 | 0.915 | 0.365 | | | 0.848 | 0.401 | -1.261 | 0.215 | | 1.638 | 0.110 | -0.941 | 0.352 |
|  | Snags | -0.592 | | 0.557 | 1.590 | 0.119 | | | -0.085 | 0.932 | 0.026 | 0.980 | | -0.083 | 0.934 | 0.344 | 0.732 |
|  | Marginal R^2^ | 0.119 | | | 0.146 | | | | 0.108 | | 0.165 | | | 0.129 | | 0.156 | |
| *P. sylvestris* | Canopy (Open) | 0.166 | 0.869 | | 0.016 | | | 0.987 | 1.837 | 0.073 | -1.739 | | 0.089 | 1.803 | 0.078 | 1.726 | 0.091 |
|  | Trees removed | 1.206 | 0.234 | | -1.513 | | | 0.137 | -0.148 | 0.883 | 0.169 | | 0.866 | 0.282 | 0.779 | 0.391 | 0.697 |
|  | Snags + Logs | -0.151 | 0.881 | | 0.340 | | | 0.735 | -0.257 | 0.799 | -0.186 | | 0.853 | 0.237 | 0.813 | 0.440 | 0.662 |
|  | Habitat trees | 0.630 | 0.532 | | -0.453 | | | 0.653 | -1.186 | 0.242 | -0.787 | | 0.435 | -0.881 | 0.383 | 1.252 | 0.217 |
|  | Crowns | **2.079** | **0.043*** | | 0.714 | | | 0.479 | -1.951 | 0.057 | 0.367 | | 0.715 | -1.702 | 0.095 | 0.235 | 0.815 |
|  | Logs | 0.959 | 0.343 | | 0.811 | | | 0.422 | -0.663 | 0.511 | -0.399 | | 0.692 | -0.154 | 0.879 | 0.781 | 0.439 |
|  | Snags | 0.348 | 0.729 | | 0.564 | | | 0.575 | -1.192 | 0.239 | -0.466 | | 0.643 | -1.126 | 0.266 | 0.655 | 0.516 |
|  | Marginal R^2^ | 0.044 | | | 0.094 | | | | 0.112 | | 0.057 | | | 0.112 | | 0.057 | |

**Table S3.** Test statistics and *p*-values from fitting a beta regression model (*gam* function, *mgcv* package) on mass loss in response to canopy cover and deadwood enrichment and for each order of *q* and for each host tree species separately. The reference level for canopy cover is stated in parentheses. The reference level for the deadwood enrichment was patches where only stumps were left remaining. Significant effects are indicated with asterisks (* *p* < 0.05, ** *p* < 0.01, ***  *p* < 0.001).

|  | Fixed effects | *q* = 0, rare | | *q* = 1, common | | *q* = 2, dominant | |
| --- | --- | --- | --- | --- | --- | --- | --- |
|  |  | **z value** | **Pr(>\|z\|)** | **z value** | **Pr(>\|z\|)** | **z value** | **Pr(>\|z\|)** |
| *F. sylvatica* | Canopy (Open) | -0.094 | 0.925 | -0.004 | 0.997 | 0.069 | 0.945 |
|  | Trees removed | **2.559** | **0.011*** | **2.445** | **0.014*** | **2.376** | **0.018*** |
|  | Snags + Logs | 1.749 | 0.080 | 1.656 | 0.098 | 1.532 | 0.126 |
|  | Habitat trees | 0.906 | 0.365 | 0.788 | 0.431 | 0.592 | 0.554 |
|  | Crowns | **2.900** | **0.004**** | **2.794** | **0.005**** | **2.859** | **0.004**** |
|  | Logs | 1.506 | 0.132 | 1.487 | 0.137 | 1.403 | 0.160 |
|  | Snags | 0.610 | 0.542 | 0.574 | 0.566 | 0.461 | 0.645 |
|  | Alpha diversity | -0.391 | 0.696 | -0.935 | 0.350 | -1.385 | 0.166 |
|  | **Deviance explained** | 27.7% | | 38.4% | | 28.7% | |
|  | Canopy (Open) | -0.319 | 0.750 | -0.215 | 0.830 | -0.231 | 0.817 |
|  | Trees removed | **2.190** | **0.028*** | **2.470** | **0.014*** | **2.479** | **0.013*** |
|  | Snags + Logs | 1.701 | 0.089 | 1.716 | 0.086 | 1.677 | 0.093 |
|  | Habitat trees | 0.817 | 0.414 | 0.813 | 0.416 | 0.799 | 0.424 |
|  | Crowns | **2.964** | **0.003**** | **2.929** | **0.003**** | **2.913** | **0.004**** |
|  | Logs | 1.645 | 0.100 | 1.555 | 0.120 | 1.544 | 0.123 |
|  | Snags | 0.571 | 0.568 | 0.549 | 0.583 | 0.476 | 0.634 |
|  | Beta diversity (PC1) | 0.880 | 0.379 | 0.343 | 0.731 | 0.441 | 0.659 |
|  | **Deviance explained** | 25.3% | | 27.7% | | 28.0% | |
|  | Canopy (Open) | -0.167 | 0.867 | -0.211 | 0.833 | -0.162 | 0.871 |
|  | Trees removed | **2.578** | **0.010*** | **2.605** | **0.009**** | **2.550** | **0.011*** |
|  | Snags + Logs | 1.642 | 0.101 | 1.592 | 0.111 | 1.731 | 0.084 |
|  | Habitat trees | 0.845 | 0.398 | 0.887 | 0.375 | 0.911 | 0.362 |
|  | Crowns | **2.856** | **0.004**** | **2.899** | **0.004**** | **2.920** | **0.004**** |
|  | Logs | 1.564 | 0.118 | 1.595 | 0.111 | 1.546 | 0.122 |
|  | Snags | 0.666 | 0.506 | 0.704 | 0.481 | 0.661 | 0.509 |
|  | Beta diversity (PC2) | -0.167 | 0.867 | -0.497 | 0.619 | 0.075 | 0.940 |
|  | **Deviance explained** | 26.7% | | 26.5% | | 26.8% | |
| *P. sylvestris* | Canopy (Open) | 0.500 | 0.617 | 0.526 | 0.599 | 0.734 | 0.463 |
|  | Trees removed | 0.528 | 0.598 | 0.544 | 0.587 | 0.523 | 0.601 |
|  | Snags + Logs | 0.986 | 0.324 | 0.713 | 0.476 | 0.602 | 0.547 |
|  | Habitat trees | -1.754 | 0.079 | -1.406 | 0.160 | -1.312 | 0.190 |
|  | Crowns | -0.276 | 0.782 | 0.123 | 0.902 | 0.262 | 0.793 |
|  | Logs | -0.411 | 0.681 | -0.564 | 0.573 | -0.489 | 0.625 |
|  | Snags | -0.294 | 0.769 | -0.163 | 0.871 | 0.131 | 0.896 |
|  | Alpha diversity | -1.091 | 0.275 | -1.648 | 0.099 | -1.491 | 0.136 |
|  | **Deviance explained** | 35.9% | | 38.8% | | 39.8% | |
|  | Canopy (Open) | 0.464 | 0.642 | 0.555 | 0.579 | 0.574 | 0.566 |
|  | Trees removed | 0.471 | 0.637 | 0.616 | 0.538 | 0.472 | 0.637 |
|  | Snags + Logs | 1.282 | 0.200 | 1.100 | 0.271 | 0.959 | 0.338 |
|  | Habitat trees | -1.069 | 0.285 | -1.696 | 0.090 | -1.745 | 0.081 |
|  | Crowns | 0.154 | 0.877 | -0.318 | 0.750 | -0.352 | 0.725 |
|  | Logs | -0.030 | 0.976 | -0.208 | 0.835 | -0.256 | 0.798 |
|  | Snags | 0.885 | 0.376 | 0.229 | 0.819 | 0.259 | 0.796 |
|  | Beta diversity (PC1) | 1.411 | 0.158 | -0.521 | 0.602 | -0.802 | 0.422 |
|  | **Deviance explained** | 39.9% | | 37.0% | | 38.1% | |
|  | Canopy (Open) | 0.751 | 0.453 | 0.003 | 0.997 | 0.325 | 0.745 |
|  | Trees removed | 0.485 | 0.628 | 0.745 | 0.456 | 0.967 | 0.334 |
|  | Snags + Logs | 1.414 | 0.157 | 1.032 | 0.302 | 1.575 | 0.115 |
|  | Habitat trees | -1.039 | 0.299 | -1.559 | 0.119 | -1.382 | 0.167 |
|  | Crowns | -0.081 | 0.936 | 0.325 | 0.745 | 0.383 | 0.702 |
|  | Logs | -0.131 | 0.896 | -0.087 | 0.931 | 0.039 | 0.969 |
|  | Snags | 0.395 | 0.693 | 0.560 | 0.575 | 0.515 | 0.607 |
|  | Beta diversity (PC2) | 1.527 | 0.127 | **2.588** | **0.010*** | **-2.381** | **0.017*** |
|  | **Deviance explained** | 40.2% | | 43.8% | | 42.4% | |
